# Integration of Multi-Scale Profiling and Machine Learning Reveals the Prognostic Role of Extracellular Matrix-Related Cancer-Associated Fibroblasts in Lung Adenocarcinoma

**DOI:** 10.7150/ijms.113580

**Published:** 2025-06-12

**Authors:** Ziyi Chen, Mengyuan Chen, Changqing Yang, Jiajing Wang, Yuan Gao, Yuanying Feng, Dongqi Yuan, Peng Chen

**Affiliations:** 1 Tianjin Medical University Cancer Institute and Hospital, National Clinical Research Center for Cancer, Key Laboratory of Cancer Prevention and Therapy, Tianjin, 300060, China.; 2 Tianjin's Clinical Research Center for Cancer, Department of Thoracic Oncology, Tianjin Lung Cancer Center, Tianjin Cancer Institute & Hospital, Tianjin Medical University, Tianjin, 300060, China.; 3 Department of Nutrition, Tianjin Medical University Cancer Institute and Hospital, Tianjin, China.; 4 Respiratory Department, Tianjin Medical University General Hospital, Tianjin, 300052, China.

**Keywords:** cancer associated fibroblasts, macrophages, lung adenocarcinoma, tumor microenvironment, machine-learning

## Abstract

Lung adenocarcinoma (LUAD) remains a leading cause of cancer mortality, necessitating novel therapeutic targets and prognostic strategies. This study investigates the role of extracellular matrix cancer-associated fibroblasts (eCAFs) and their interaction with SPP1+ macrophages in LUAD progression and prognosis. Utilizing single-cell RNA sequencing from 15 LUAD tumors and integrating multi-cohort transcriptomic data (TCGA, GSE31210, GSE72094), we identified eCAFs as a dominant CAF subtype in advanced-stage tumors and high-grade pathological subtypes, correlating with poor patient survival. Similarly, SPP1+ macrophages exhibited increased abundance in advanced tumors and adverse prognosis. Pseudotime trajectory analysis revealed eCAFs as an evolutionary endpoint in CAF differentiation, associated with extracellular matrix remodeling pathways (COLLAGEN, FN1). Cell-cell communication analysis highlighted eCAFs-SPP1+ macrophage interactions via COL1A1-CD44 and COL1A2-CD44 ligand-receptor pairs, suggesting a mechanism for immune-excluded microenvironments. A prognostic model incorporating 28 eCAFs-related genes, validated through 101-machine learning algorithms, effectively stratified patients into high- and low-risk groups across cohorts. This study underscores eCAFs as key drivers of LUAD progression and proposes their interplay with SPP1+ macrophages as a therapeutic target. The developed prognostic signature offers clinical utility for risk stratification, though further experimental validation is warranted. These findings advance understanding of stromal-immune crosstalk in LUAD and highlight ECM remodeling as a critical pathway in tumor evolution.

## Introduction

In recent years, cancer has emerged as a major global public health challenge, imposing disparate healthcare burdens across nations while remaining as a leading cause of mortality worldwide 1. Lung cancer persistently maintains its status as one of the most lethal malignancies in both incidence and mortality rates 1-3. Non-small cell lung cancer (NSCLC) accounts for the predominant histological subtype, with adenocarcinoma representing its most frequent variant 4, 5. Notwithstanding advances in therapeutic target identification for lung adenocarcinoma, persistent clinical challenges remain, stemming from intratumoral heterogeneity, interpatient variability, and the development of therapeutic resistance 6, 7. Furthermore, lung cancer is frequently diagnosed at advanced stages, contributing to unfavorable patient prognosis 8. Therefore, considering these established challenges, it is essential to identify new therapeutic targets that influence patient prognosis in lung adenocarcinoma and to evaluate novel prognostic approaches, thereby expanding clinical benefits for patients.

Cancer-associated fibroblasts (CAFs) represent pivotal stromal constituents within the tumor microenvironment, demonstrating marked heterogeneity across cancer types and tissue niches. CAFs receive bidirectional signaling from tumor cells, immune populations, and other stromal elements in the tumor microenvironment, thereby regulating the growth, metastasis, and therapeutic resistance of the tumor 9. Although previous studies have primarily focused on the signaling and interactions between CAFs and tumor cells, increasing evidence suggests that the reciprocal effects between CAFs and the tumor immune microenvironment also play a crucial role in tumor development 10. Therefore, a deeper understanding of the mechanisms linking CAFs and immune cells may provide new strategies for subsequent targeted immunotherapy, offering fresh insights into precision treatment for cancer patients.

CAFs are highly heterogeneous, composed of different subpopulations with complex cellular origins and diverse biological functions, playing distinct roles in tumors 11. In various cancer types, CAFs can mainly be classified into antigen-presenting CAFs (apCAFs), myofibroblastic CAFs (myCAFs), and inflammatory CAFs (iCAFs) 9. However, with the advancement of single-cell technologies, we are now able to identify different cellular subpopulations with greater resolution 12. In gastric cancer, extracellular matrix CAFs (eCAFs) identified via the expression of POSTN demonstrate pro-invasive properties 13. Furthermore, it has been reported that these extracellular matrix-related CAFs interact closely with immune cells, particularly macrophages 14. Nevertheless, the biological significance of eCAFs in lung adenocarcinoma remains underexplored. Therefore, this study aims to rigorously identify eCAFs while ensuring data reliability and further investigate their detailed biological functions and their interactions with macrophages.

We found that eCAFs account for a higher proportion of CAFs in advanced tumors and in pathological subtypes with higher malignancy, and the abundance level of eCAFs is closely associated with poor prognosis in lung adenocarcinoma patients. Lineage trajectory analysis demonstrated that eCAF development is evolutionarily linked to tumor progression cascades, positioning this subpopulation as a central mediator of malignant transformation. A similar pattern was also observed in SPP1+ macrophages. Mechanistically, we identified ligand-receptor axes (COL1A1-CD44 and COL1A2-CD44) mediating functional cross-talk between eCAFs and SPP1+ macrophages. Additionally, we observed high activity of eCAFs in the COLLAGEN signaling pathway and FN1 signaling pathway. Finally, we established a novel prognostic model for lung adenocarcinoma patients using 101-combination machine learning algorithms. In conclusion, the prognostic value of eCAFs and their potential as a key therapeutic strategy are critical for lung adenocarcinoma patients.

## Materials and Methods

### Data acquisition and pre-processing

Derived from the Gene Expression Omnibus Series (GEO), the GSE131907 dataset comprises 58 single-cell RNA sequencing samples obtained from different lung adenocarcinoma patients. These samples include primary tumors, lymph nodes, brain metastases, pleural effusion, as well as normal lung tissue and lymph nodes. Subsequently, we selected 15 lung adenocarcinoma tumor samples from the dataset for further analysis. Bulk RNA transcriptome data and clinical data were collected from the LUAD cohort of The Cancer Genome Atlas (TCGA, https://portal.gdc.cancer.gov/) using the 'TCGAbiolinks' package 15, as well as the GSE72094 and the GSE31210 dataset from GEO database. Single-cell RNA sequencing data were processed and analyzed utilizing the Seurat package (v4.3.0) based on the R programming language (v4.3.3). In order to ensure the quality and reliability of single-cell transcriptomic data, the routine quality control was performed on each dataset with the following criteria applied: nFeature_RNA ≥ 200, nFeature_RNA ≤ 10,000, nCount_RNA ≥ 100, nCount_RNA ≤ 150,000, and percent_mito ≤ 20. The potential doublets were removed using the R software package DoubletFinder (version 2.0.3) 16. Harmony (version 1.2.0) 17 was implemented for the integration of heterogeneous sample datasets and the elimination of batch effects. Sctransform (version 0.3.5) 18 was subsequently employed for data normalization and the removal of technical variation sources.

### Downstream analysis of sc-RNA transcriptome sequencing data

The biological functional markers used to annotate different cell populations were derived from the CellMarker 2.0 online database 19 and published literature. The Dotplot and Vinplot functions from the Seurat (version 4.3.0) package were used to visualize the expression levels of different biological markers across various cell populations. The FeaturePlot function was used to visually depict the distribution of cell populations and ensure the reliability of annotations for different cell groups. The FindMarkers function was employed to identify characteristic differentially expressed genes of eCAFs and CAFs. Combined alluvial diagram and stacked bar chart were conducted with the R package ggalluvial (version 0.12.5). The R package ramcharts4 (version 1.6.0) was used to create a pie chart visualizing the proportion of different cells. The ggplot2 package (version 3.5.1) was used to enhance the clarity and aesthetic appeal of the results.

### Identification of survival phenotype-associated cells

We systematically identified cells associated with survival phenotypes by integrating TCGA bulk RNA-seq datasets with matched clinical survival data. Through the implementation of the Scissor R package (version 2.0.0) 20, we established quantitative associations between gene expression profiles and single-cell transcriptomic signatures, while integrating survival phenotypes into a network-constrained sparse regression framework. This computational strategy enabled the detection of putative survival-relevant cellular subsets. Notably, the Scissor+ cells subpopulation was considered to be correlated with poor clinical outcomes.

### Survival analysis

To further evaluate the association between eCAF subsets and SPP1+ macrophage subsets with patient survival, we screened lung adenocarcinoma datasets containing both bulk transcriptomic profiles and survival information. Ultimately, three independent cohorts were included: 503 samples from the TCGA-LUAD cohort, 226 samples from the GSE31210 cohort, and 398 samples from the GSE72094 cohort. The CIBERSORTx platform 21 was employed to integrate single-cell RNA sequencing data with bulk transcriptomic data. Using the CIBERSORTx deconvolution algorithm in absolute mode 22, we estimated the relative proportions of specific cellular subsets by leveraging reference gene expression signatures derived from single-cell RNA data. Patients were subsequently stratified into high-infiltration and low-infiltration groups based on the relative abundance of corresponding cellular subsets. Kaplan-Meier (KM) survival curves were generated for selected cell subpopulations, with a p-value < 0.05 indicating statistical significance.

### Cell pseudo-time trajectory analysis

To further elucidate the potential developmental trajectories and evolutionary characteristics of cellular subpopulations, we employed the SCP package (https://github.com/zhanghao-njmu/SCP) to infer pseudo-time developmental trajectories using the Slingshot algorithm. Concurrently, we conducted comprehensive analyses of dynamic biological functional alterations within these cellular populations, encompassing both gene expression profiling and Gene Ontology (GO) enrichment analysis. Meanwhile, the GSVA package (version 1.50.5) and the clusterProfiler package (version 4.12.2) were used to analyze hallmark pathway activity across different cell populations.

### Cell to cell communication

Cell-to-cell communication analysis was performed utilizing CellChat (version 1.6.1) 23 with the CellChatDB.human database for analytical purposes. To further investigate potential cellular communication between eCAFs and SPP1+ macrophage in lung adenocarcinoma, we systematically identified ligand-receptor interactions by designating SPP1+ macrophage cells as the target population and CAFs as the source population. These interactions were subsequently visualized through bubble plot. Additionally, we performed heatmap analysis to delineate key biological signaling pathways associated with these cellular interactions.

### Machine-learning framework

Machine learning workflows were implemented with the Mime1 package (version 0.0.0.9) 24, a comprehensive toolkit integrating 101-machine learning algorithms for model training, validation, and evaluation. The FindMarkers function was systematically applied to identify signature genes distinguishing CAFs from other cell types, followed by intra-CAFs differential analysis to delineate the eCAFs-specific signatures within the CAFs population. Intersection of these comparative gene sets revealed 289 consensus genes co-expressing both pan-CAFs and eCAFs signatures. Subsequently, stringent thresholds (log2FC ≥ 1, adjusted p-value ≤ 0.05) were implemented to screen 888 upregulated genes in the tumor tissues of TCGA-LUAD cohort using limma package (version 3.58.1). Ultimately, 28 core molecular signatures were prioritized for integration into the machine learning workflow. The parameter configuration was established such that only genes demonstrating statistically significant associations in univariate Cox proportional hazards analysis were incorporated into subsequent analytical workflows. The TCGA-LUAD cohort served as the primary training dataset (dataset1), while the GSE31210 and GSE72094 cohorts were designated as independent validation datasets (dataset2 and dataset3, respectively).

### Statistics

All statistical analyses were performed using R software (version 4.3.3). p < 0.05 was considered statistically significant.

## Results

### Detailed annotation of CAFs, macrophages in single-cell data

**Figure 1:**
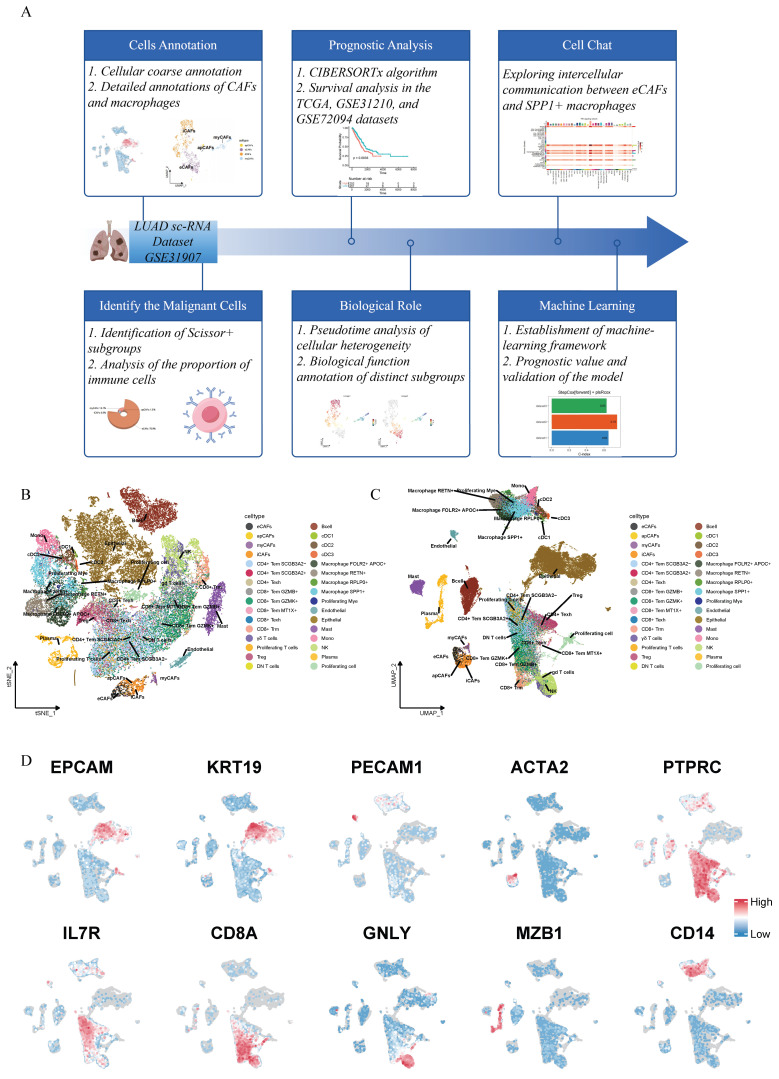
** Workflow chart and the annotation of single-cell transcriptome data of lung adenocarcinoma.** (A) Workflow chart of the study. (B) t-Distributed Stochastic Neighbor Embedding (t-SNE)-based (right) dimensionality reduction map. (C) Uniform Manifold Approximation and Projection (UMAP)based dimensionality reduction map. The cell cluster annotations are marked with different colors. (D) Schematic illustration of markers used for preliminary cell clustering in UMAP: EPCAM and KRT18 for epithelial cells; PECAM1 for endothelial cells; ACTA2 for fibroblasts; PTPRC for immune cells; IL7R for Th cells; CD8A for Tc cells; GNLY for NK cells; MZB1 for B cells; and CD14 for myeloid cells.

**Figure 2:**
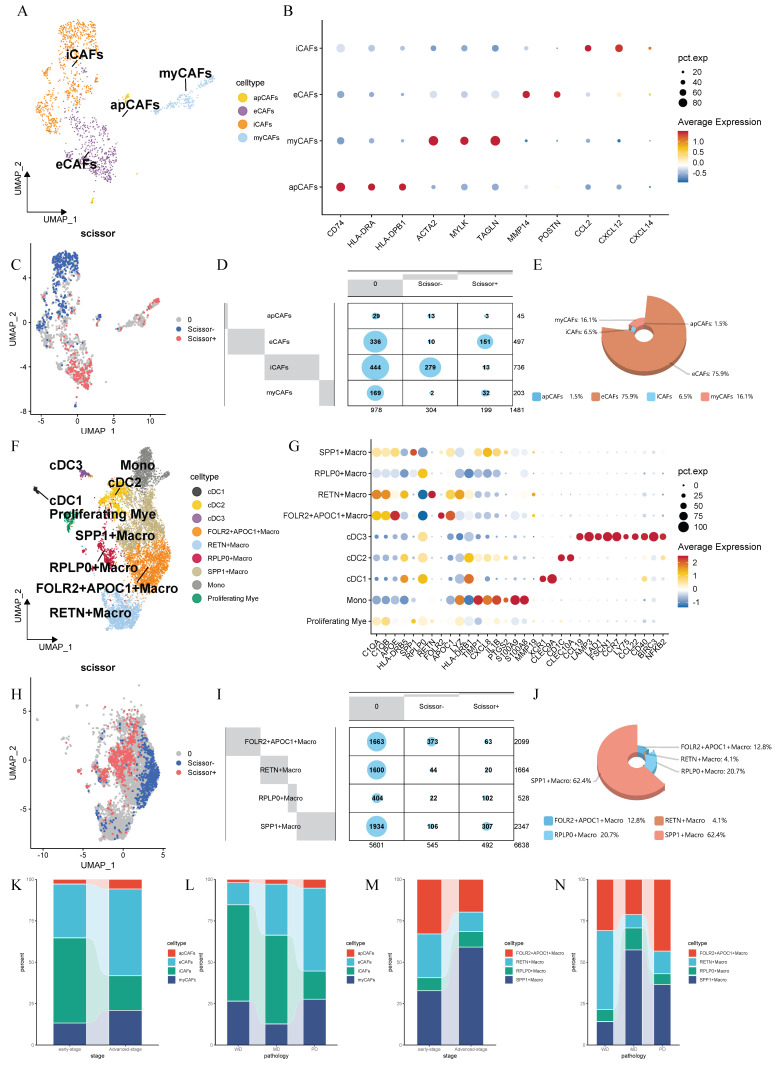
** Detailed annotations, prognostic phenotypic associations and cellular proportion analysis of CAFs and macrophages.** (A) UMAP-based dimensionality reduction map of fibroblast subtypes, including eCAFs, iCAFs, myCAFs, and apCAFs. Different cell clusters are represented by distinct colors. (B) Dotplot showing the expression of representative marker genes in fibroblast subtypes. The color represents the average expression level, and the dot size indicates the percentage of cells expressing the gene. (C) UMAP plot displaying the distribution of Scissor+ and Scissor- fibroblasts. (D) Contingency table showing the distribution of Scissor+ and Scissor- cells across fibroblast subtypes. (E) 3D pie chart showing the proportion of fibroblast subtypes among Scissor+ cells. (F) UMAP plot of myeloid cells. (G) Dotplot showing the expression of representative marker genes in myeloid cells. (H) UMAP plot displaying the distribution of Scissor+ and Scissor- macrophages. (I) Contingency table showing the distribution of Scissor+ and Scissor- cells across macrophage subtypes. (J) 3D pie chart showing the proportion of macrophage subtypes among Scissor+ cells. (K-N) Stacked bar plots showing the distribution of CAFs (K, L) and macrophage (M, N) subtypes across different disease stages and pathological conditions.

As shown in the workflow diagram in Figure 1A, this tudy aims to elucidate the biological functions of cancer-associated fibroblasts (CAFs) in lung adenocarcinoma (LUAD) and investigate their interplay with macrophages in the tumor microenvironment. Rigorous cellular annotation was therefore necessarily performed. Following batch quality control, batch effect correction, and doublet removal, high-quality single-cell data were obtained. Optimal dimensionality reduction clustering resolution was determined using clustree analysis, with subsequent visualization through t-Distributed Stochastic Neighbor Embedding (t-SNE) and Uniform Manifold Approximation and Projection (UMAP) algorithms (Figure 1B-C). Primary cell classification was achieved using classic markers: EPCAM/KRT19 for epithelial cells, PECAM1/ACTA2 for stromal cells and PTPRC for immune cells. Subpopulation identification revealed T helper cells (IL7R+), cytotoxic T lymphocytes (CD8A+), natural killer cells (GNLY+), plasma cells (MZB1+), and myeloid cells (CD14+) (Figure 1D). Given the functional heterogeneity of CAFs, we performed secondary clustering to define four distinct subtypes: antigen-presenting CAFs (apCAFs), myofibroblastic CAFs (myCAFs), extracellular matrix CAFs (eCAFs), and inflammatory CAFs (iCAFs) (Figure 2A). These subtypes exhibited characteristic expression profiles: apCAFs demonstrated up-regulated MHC class II molecules; myCAFs expressed myofibroblastic molecules markers (ACTA2, MYLK, TAGLN); eCAFs showed extracellular matrix remodeling signatures (MMP14, POSTN); while iCAFs were enriched in inflammatory factors (CCL2, CXCL12, CXCL14) (Figure 2B). Within the myeloid compartment, we identified four distinct macrophage subpopulations through the selection of top-ranked and functionally specific marker genes: SPP1+ macrophages, RPLP0+ macrophages, RETN+ macrophages, and FOLR2+APOC1+ macrophages (Figure 2F-G).

### ECAFs and SPP1+ macrophages demonstrated a significant association with poor prognostic outcomes

**Figure 3:**
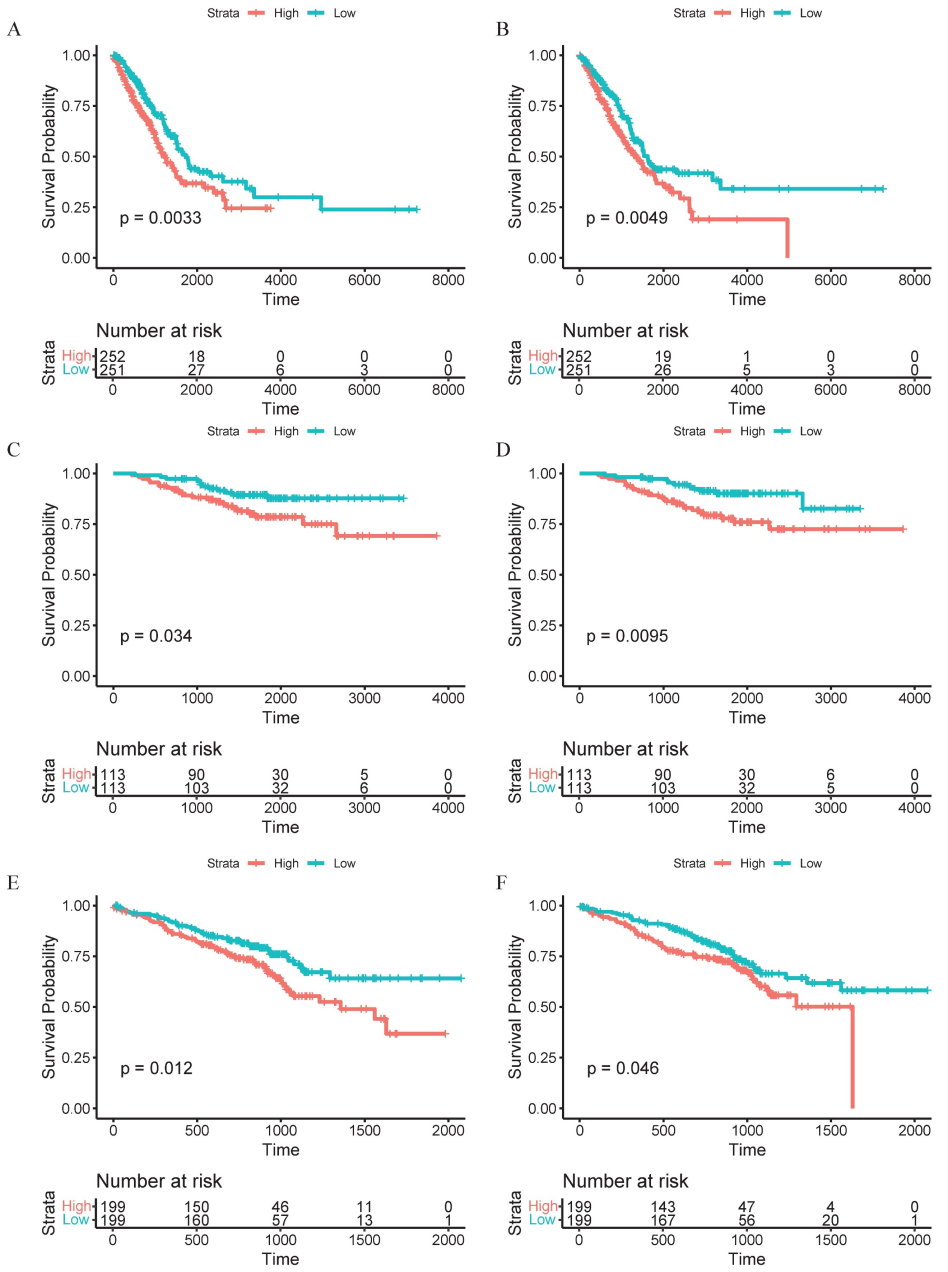
** High infiltration of eCAFs and SPP1+ macrophages suggests a poor prognosis.** (A, C, E) Kaplan-Meier curves for eCAFs of high infiltration level and low infiltration level. (B, D, F) Corresponding survival analyses for SPP1+ macrophages. (A-B) TCGA cohort; (C-D) GSE31210 cohort; (E-F) GSE72094 cohort. Log-rank p-values are indicated for each comparison. Numbers for "High" and "Low" groups are shown below each survival plot. Statistical significance was defined as p < 0.05.

To integrate bulk transcriptomic data with single-cell data, we employed the Scissor algorithm to further identify potential associations between cells of interest and patient prognosis 20. After collecting bulk RNA sequencing data and survival data from TCGA, we identified Scissor+ cells associated with poor survival outcomes in both CAFs and macrophages (Figure 2C, H). Researchers found that eCAFs constitute a significant component of Scissors+ cells, accounting for 75.9% of the subpopulation proportion in Scissors+ cells, while iCAFs represent the majority of Scissors- cells (Figure 2D-E). Within the macrophage population, we found that SPP1+ macrophages constitute a significant component of Scissors+ cells, accounting for 62.4% of the cellular subpopulation proportion in Scissors+ cells (Figure 2I-J). Therefore, we reasonably conclude that both eCAFs and SPP1+ macrophages are closely associated with the poor survival outcomes. To validate this association, we implemented the CIBERSORTx computational algorithm to quantify eCAF and SPP1+ macrophage infiltration levels across three independent cohorts (TCGA, GSE31210, GSE72094). Patients were then divided into high-infiltration and low-infiltration groups based on the median values of infiltration abundance level. It's worth noting that racial background did not substantially affect the infiltration levels of these cell subsets (Supplementary Tables 1 and 2). Subsequent Kaplan-Meier survival analysis revealed that in the TCGA cohort (p=0.0033, Figure 3A), GSE31210 cohort (p=0.034, Figure 3C), and GSE72094 cohort (p=0.012, Figure 3E), the high-infiltration group of eCAFs exhibited significantly worse survival outcomes compared to the low-infiltration group. Similarly, the high-infiltration group of SPP1+ macrophages demonstrated poorer survival in the TCGA cohort (p=0.0049, Figure 3B), GSE31210 cohort (p=0.0095, Figure 3D), and GSE72094 cohort (p=0.046, Figure 3F).

### The proportion of eCAFs and SPP1+ Macrophages varies with the stage and pathological classification

Tumor cells typically alter the surrounding microenvironment during progression, often accompanied by changes in the cellular composition. Building upon the established prognostic significance of eCAFs and SPP1+ macrophages, we performed systematic quantification of their temporal-spatial distribution patterns across tumor stages and histological differentiation grades. We observed that in advanced-stage LUAD, the proportions of both eCAFs and SPP1+ macrophages increased with the progression of tumor (Figure 2K, M). In pathological classification, eCAFs proportion increased with decreasing differentiation (Figure 2L). However, this pattern was absent in SPP1+ macrophages, which exhibited peak abundance in moderately-differentiated tumors and lowest levels in well-differentiated tumors (Figure 2N).

### The intrinsic developmental trajectories of CAFs and macrophages

**Figure 4:**
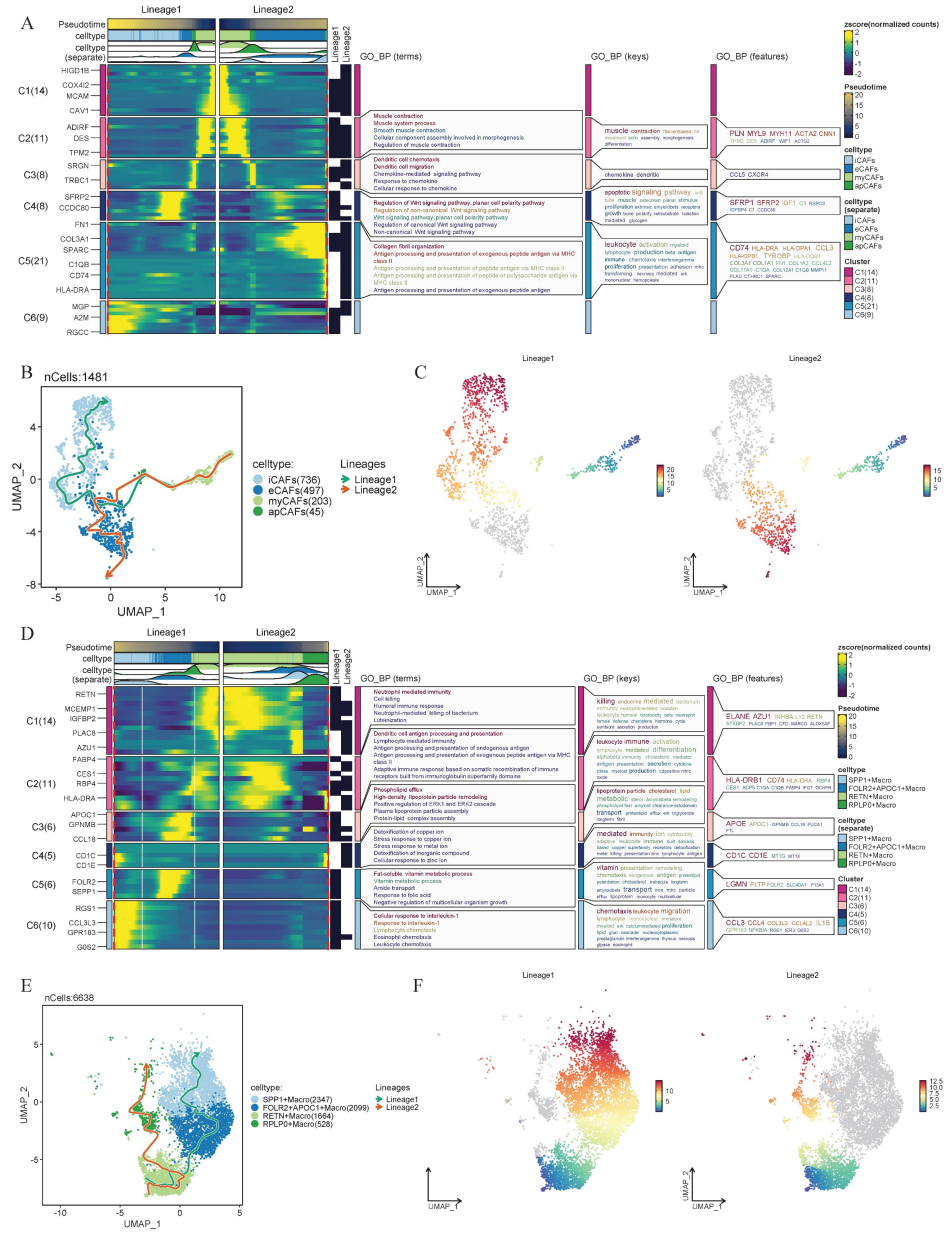
** Integrated analysis of cellular heterogeneity, differentiation trajectories, and functional annotations.** (A) Changes in gene expression of CAFs in Lineage 1 and Lineage 2, with functional enrichment results of GO_BP for different gene lists shown on the right. (B-C) Pseudo-time developmental trajectories of CAFs. Lineage1 and Lineage2 denote distinct differentiation trajectories inferred from pseudotime analysis. (D) Changes in gene expression of macrophages in Lineage 1 and Lineage 2. (E-F) Pseudo-time developmental trajectories of macrophages.

Considering the strong association between eCAFs and SPP1+ macrophages within their respective cellular subpopulations and their relevance to prognosis and tumor progression, researchers performed pseudotime analysis to further explore their intrinsic lineage evolution processes. We found that both CAFs and macrophages exhibit distinct trends in cellular development and evolution. Based on pseudotime progression, two lineage evolution processes were observed in CAFs. In Lineage1, CAFs demonstrated a differentiation trajectory from myCAFs toward iCAFs. In Lineage2, eCAFs served as the developmental endpoint, with CAFs exhibiting a differentiation trajectory from myCAFs to eCAFs (Figure 4B-C). Simultaneously, the heatmap revealed the biological functional evolution during the developmental process. In Lineage2, the expression changes of key genes demonstrated a functional transition of CAFs from muscle contraction-related activities to immune regulation, followed by Wnt signaling pathway modulation, and ultimately to extracellular matrix remodeling (Figure 4A). Within the macrophages, we also observed two distinct intrinsic lineage differentiation trajectories. In the Lineage1 of macrophages, RETN+ macrophages progressively differentiated into FOLR2+APOC1+ macrophages and ultimately evolved into SPP1+ macrophages. In the Lineage2, RETN+ macrophages exhibited a differentiation trajectory toward RPLP0+ macrophages (Figure 4E-F). Notably, in the functional evolution Lineage1, macrophages transitioned from cellular immunity and antigen presentation functions to lipoprotein-associated functions, subsequently shifted toward signaling regulation and immune cell recruitment (Figure 4D).

### Functional enrichment analysis

**Figure 5:**
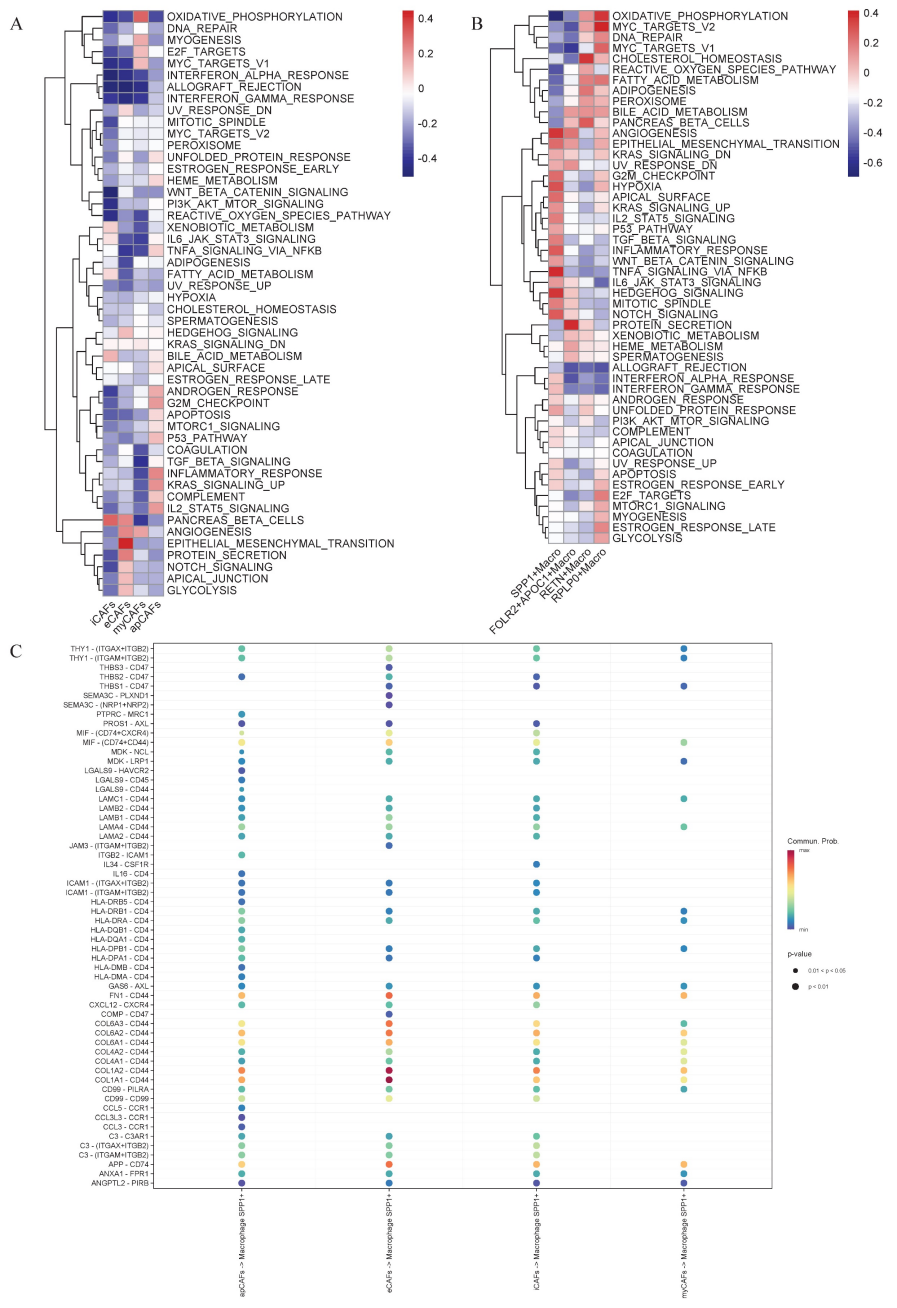
** Identification of potential receptor-ligands for eCAFs-SPP1+ macrophage communication and the functional enrichment analysis of CAFs and macrophages.** The hallmark pathway enrichment score of different CAFs subpopulation cells (A) and macrophages subpopulation cells (B) were illustrated by heat map. (C) Dot plot illustrating the interactions of CAFs to SPP1+ macrophages.

To further characterize the biological features of eCAFs and SPP1+ macrophages, we performed Gene Set Variation Analysis (GSVA) functional enrichment analysis on the signature gene sets. Compared to other CAF subtypes, eCAFs exhibited significant enrichment in pathways including angiogenesis, epithelial-mesenchymal transition, protein secretion, Notch signaling, apical junction, and glycolysis (Figure 5A). In the macrophages population, SPP1+ macrophages exhibited significant enrichment in pathways including angiogenesis, epithelial-mesenchymal transition, inflammatory response, G2/M checkpoint, TNF-α signaling via NF-κB, Hedgehog signaling, and Notch signaling (Figure 5B).

### Intercellular communication among the various components of the tumor microenvironment

**Figure 6:**
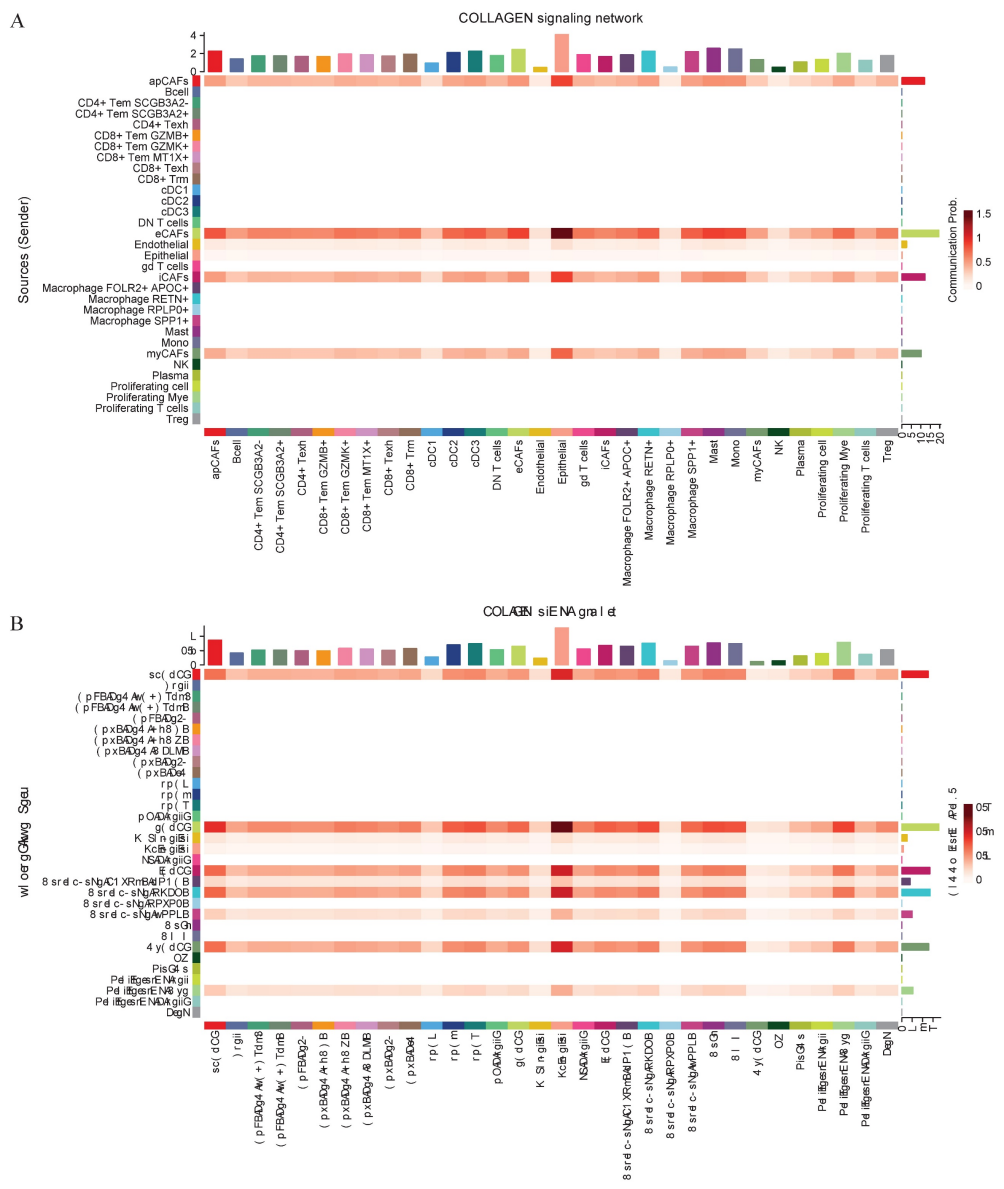
** Cell-cell communication networks mediated by COLLAGEN and FN1 signaling pathways.** CellChat analysis depicting intercellular communication probabilities across diverse cell populations. Collagen Signaling Network (A) and FN1 Signaling Network (B) are illustrated by heatmaps. The sender cell types are indicated, and the strength of communication from sender to receiver is represented by the color intensity.

Intercellular communication within the tumor microenvironment constitutes a fundamental driver of tumor initiation, progression, and therapy resistance. Investigating these interactions not only reveals the evolutionary logic of tumors but also provides deeper insights into their dynamic adaptability and heterogeneity. Given the close association between eCAFs, SPP1+ macrophages, and poor prognosis, researchers further conducted an intercellular communication analysis. Bubble plot analysis revealed close communication between eCAFs and SPP1+ macrophages, with significant enrichment in the COL1A1-CD44 and COL1A2-CD44 pathways (Figure 5C). Subsequent analysis of selected functional pathways demonstrated that eCAFs exhibited strong communication activity across multiple receptor-ligand pairs within the COLLAGEN signaling network, indicating their pivotal role in this network. Furthermore, this pathway showed the most significant enrichment in the communication between eCAFs and epithelial cells (Figure 6A). In the FN1 signaling network, we observed high activity between four distinct types of CAFs and other cells. Notably, eCAFs showed the most pronounced activity, particularly in the communication between eCAFs and epithelial cells (Figure 6B).

### Identification of the eCAFs signatures

**Figure 7:**
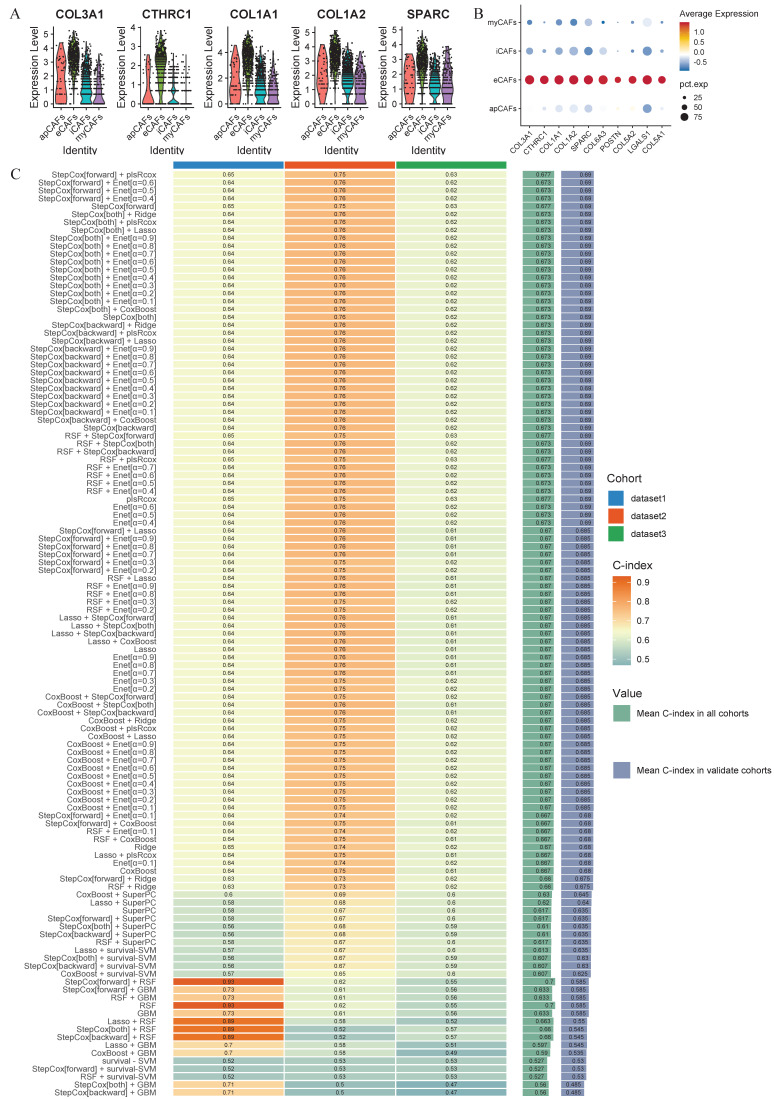
** Machine-learning framework.** (A) The expression levels of the top 5 eCAFs-related signatures across different CAF cell populations are displayed using violin plots. (B) Dotplot demonstrates the specificity of the top 10 expressed eCAFs-related genes. (C) The C-index of 101-machine learning algorithms is presented across different cohorts. The algorithm ranked first is the best-performing model. Dataset1 = TCGA, Dataset2 = GSE31210, Dataset3 = GSE72094.

Given the unique biological function and proportion of eCAFs in the LUAD tumor microenvironment, we aimed to identify a clinically valuable prognostic model through machine learning. Initially, we established specific signatures for eCAFs. This involved three sequential steps: (1) identifying characteristic signatures distinguishing CAFs from other cell types, (2) defining eCAFs-specific signatures within the CAFs population, and (3) screening tumor-upregulated differentially expressed genes in the TCGA dataset. Ultimately, the 28 specific signatures of eCAFs were incorporated into subsequent machine learning modeling. We visualized the top 5 and top 10 genes to assess their specific expression in eCAFs (Figure 7A-B).

### Construction of the machine learning model framework

**Figure 8:**
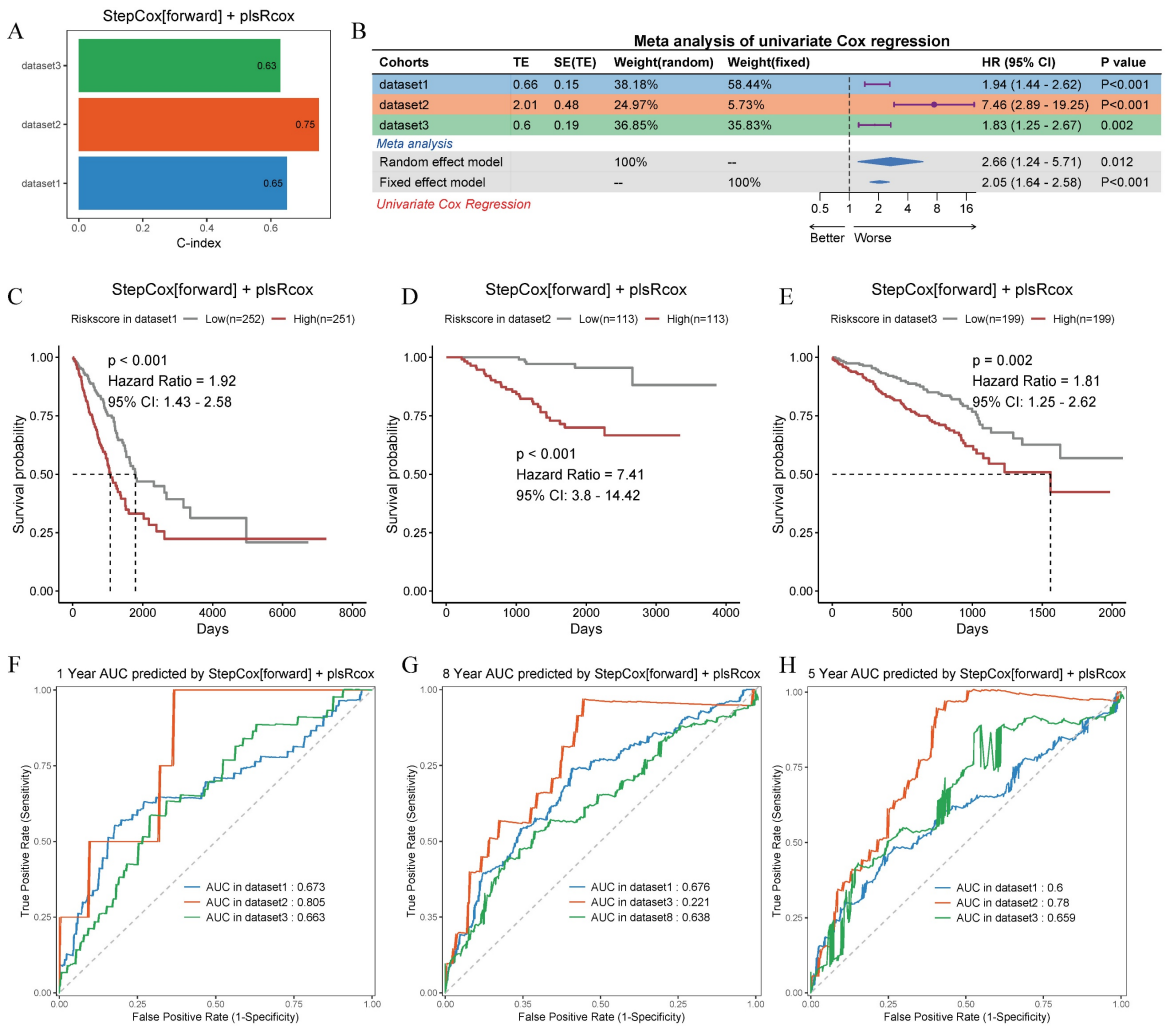
** Prognostic performance and validation of the StepCox[forward] + plsRcox risk model across multiple cohorts.** (A) Bar plot showing the C-index values for the risk model in three independent datasets. (B) Meta-analysis of the C-index using univariate Cox regression in random and fixed effect models, with percentage contributions from each dataset. Hazard ratios (HR) and 95% confidence intervals (95% CI) are on the right. (C-E) Kaplan-Meier survival curves stratified by low-risk and high-risk groups in dataset1, dataset2 and dataset3. p-values and HRs are annotated. (F-H) Time-dependent receiver operating characteristic (ROC) curves for 1-year, 3-year, and 5-year survival predictions in dataset1, dataset2 and dataset3.

After analyzing 101 machine learning model combinations, we ranked them based on the mean C-index across all cohorts to identify the best-performing modeling method (Figure 7C). The StepCox[forward] + plsRcox model demonstrated the most favorable results. The C-index of this model was 0.65 in the training set, 0.75 in the validation set GSE31210, and 0.63 in the validation set GSE72094 (Figure 8A). After dividing the patients into two subgroups based on the median risk score, the univariate Cox regression meta-analysis showed that the model was positively correlated with the risk of patient mortality (HR > 1), and all datasets demonstrated significant p-values (Figure 8B). In the meta-analysis, the HR values for the Random Effect Model and the Fixed Effect Model were 2.66 (1.24 - 5.71) and 2.05 (1.64 - 2.58), respectively. The combined results showed a significant increase in risk and the effect of risk scores remaining stable. Using the median score from the model, patients were categorized into high-risk and low-risk groups. In the training set (HR = 1.92, 95% CI: 1.43-2.58, p < 0.001), the GSE31210 validation set (HR = 7.41, 95% CI: 3.8-14.42, p < 0.001), and the GSE72094 validation set (HR = 1.81, 95% CI: 1.25-2.62, p = 0.002), the high-risk group consistently demonstrated poorer prognosis (Figure 8C-E). The ROC curve analysis revealed the following AUCs: 1-year AUCs for TCGA-training set: 0.673, GSE31210: 0.805, GSE72094: 0.663; 3-year AUCs for TCGA-training set: 0.686, GSE31210: 0.771, GSE72094: 0.623; and 5-year AUCs for TCGA-training set: 0.6, GSE31210: 0.78, GSE72094: 0.659 (Figure 8F-H).

## Discussion

To our knowledge, this study is the first to systematically characterize the significant role of eCAFs in prognosis and their potential interactions with immune cells within the tumor microenvironment in lung adenocarcinoma. We integrated high-quality single-cell data from multiple samples, multi-cohort transcriptomic data, and diverse machine learning frameworks, which logically emphasize the critical role of eCAFs in lung adenocarcinoma prognosis. We delineate the infiltration levels, intrinsic lineage trajectories, functional evolution, and crosstalk between eCAFs and SPP1⁺ macrophages in the lung adenocarcinoma microenvironment, as well as their prognostic value.

While the presence of cancer-associated fibroblasts (CAFs) in tumor microenvironment is well-documented, their functional duality remains debated. Some reports have shown that CAFs directly influence many biological functions of cancer cells, such as proliferation, migration, and drug resistance, thereby being considered as pro-tumorigenic 25. However, other studies suggest that CAF subtypes with different characteristics may also exert tumor-suppressive effects 26. CAFs can either promote or suppress tumor growth, with their functional and phenotypic diversity linked to differential expression of tumor and stromal cell signals, markers, and genetic characteristics, which in turn is closely related to patient prognosis 9. This heterogeneity of CAFs is also a critical issue in our study. Single-cell technological advancements now permit precise functional characterization of CAF subtypes. This study elucidates the close correlation between eCAFs and prognosis in lung adenocarcinoma patients using single-cell and transcriptomic data, investigates the interactions of eCAFs with other components of the tumor microenvironment, explores their functional evolution and innovatively establishes a scoring signature (eCAFsRS) to assess patient prognosis.

The interplay between CAFs and immune cells has also garnered growing attention, particularly macrophages. In colorectal cancer, Qi et al. demonstrated that CAFs and SPP1⁺ macrophages collaborate to remodel the ECM, creating a fibrotic stroma that impedes lymphocyte infiltration into the tumor core 27. Similarly, You et al. reported in gastric cancer that eCAFs-derived periostin (POSTN) mediates macrophage chemoattraction through cellular crosstalk, consequently modulating responses to immune checkpoint blockade (ICB) therapy 14. These findings motivate our investigation into whether analogous mechanisms exist in lung adenocarcinoma.

Our findings suggest that the infiltration abundance of eCAFs and SPP1+ macrophages act as prognostic markers of poor patient survival. Notably, within Scissors+ cells associated with adverse survival phenotypes, eCAFs constituted the predominant CAF subtype (75.9%), while SPP1+ macrophages represented the majority (62.4%) of Scissors+ macrophages, underscoring their clinical relevance.

As tumors progress, the proportion of eCAFs within the CAF cell population increases, and the proportion of SPP1+ macrophages within the macrophage population also rises. Pathological stratification revealed a significant correlation between higher eCAFs abundance and malignant grades, whereas SPP1+ macrophage distribution showed no such association. Crucially, multi-cohort validation (TCGA, GSE31210, GSE72094) consistently demonstrated significantly shorter overall survival in high-infiltration groups for both cellular subtypes. These results collectively highlight the central role of eCAFs in driving aggressive tumor behavior and unfavorable clinical outcomes. While our study demonstrates that the infiltration levels of eCAFs and SPP1⁺ macrophages are significantly associated with patient survival across multiple LUAD cohorts, we acknowledge that these findings are derived from transcriptomic analyses and do not account for other important clinical variables such as treatment regimen, tumor stage, or comorbid conditions. Therefore, although the identified associations are biologically and prognostically relevant, future prospective studies incorporating comprehensive clinical metadata are warranted to validate and extend the clinical applicability of our findings.

The classification of CAFs reflects not simple stratification but dynamic transformation 28. To elucidate this complexity, researchers aim to further investigate whether there is an intrinsic lineage evolution process within CAFs. Pseudotemporal trajectory analysis revealed two divergent differentiation paths, with Lineage2 terminating at eCAFs as the evolutionary endpoint. While esophageal CAFs reportedly promote tumor growth via epithelial-mesenchymal transition (EMT) 29, our pathway enrichment data position eCAFs as pivotal EMT drivers in lung adenocarcinoma. Furthermore, pseudotime analysis indicates the potential presence of a developmental trajectory within the CAF subpopulation that leads to the formation of malignant phenotype-associated eCAFs. As the endpoint of this trajectory, eCAFs could be a potential marker for disease progression and poor prognosis in lung adenocarcinoma patients. Notably, analogous differentiation dynamics were observed in SPP1+ macrophages. SPP1 (Secreted Phosphoprotein 1) encodes osteopontin which is involved in various functions such as cell signaling, immune modulation, cell adhesion, and migration. Liu et al. demonstrated that SPP1⁺ macrophages form a tumor immune barrier in hepatocellular carcinoma, contributing to immunotherapy resistance 30. Other studies have also shown that SPP1+ macrophages are considered key fibrotic cells in certain chronic organ injuries 31. In functional enrichment of SPP1+ macrophages, we also observed the enrichment of malignant pathways including angiogenesis. These phenomena underscore the malignant role of SPP1+ macrophages in tumors.

Recent studies have revealed close intercellular interactions between CAFs and SPP1+ macrophages 27. This prompted our investigation into the crosstalk between eCAFs and SPP1⁺ macrophages. We identified that eCAFs exert significant effects on SPP1+ macrophages through the COL1A1-CD44 and COL1A2-CD44 ligand-receptor pairs. The two α1 chains encoded by COL1A1 and COL1A2, along with one α2 chain, constitute type I collagen, which serves as a major component of the extracellular matrix (ECM) 32. CD44, a member of the transmembrane glycoprotein family, mediates cell-ECM communication and adhesion, and has been reported to mediate critical steps in bone metastasis 33. For a long time, the extracellular matrix (ECM) was regarded as an inert framework and largely overlooked. However, it is now recognized as a highly dynamic partner of the immune system, providing not only dynamic tissue integrity but also acting as a signaling molecule that participates in and drives numerous biological responses, including interactions with immune cells 34. The density, composition, and stiffness of the ECM influence the infiltration of immune cells into the core of the tumor, thus affecting the antitumor immune response 35. It has been confirmed that dense collagen in the tumor microenvironment (TME) inhibits T cell infiltration 36. In ovarian cancer, collagens produced by fibroblasts correlate with the expression of Treg and T helper 2 cell (TH2) differentiation markers, suggesting their immune-suppressive and tumor-promoting roles 37. Fibronectin (FN1), an ECM protein, has been reported to be associated with tumorigenesis 38. Notably, we observed pronounced eCAF activity in both COLLAGEN and FN1 signaling, suggesting that eCAFs may enhance ECM density in lung adenocarcinoma and foster an immune-suppressive niche.

The application of machine learning algorithms is gaining increasing attention, as they offer powerful tools for extracting meaningful biological patterns from high-dimensional data. Compared to traditional approaches, these algorithms are often more effective in handling a wide range of complex biological tasks 39, 40. Therefore, we innovatively selected 28 eCAFs-related genes to construct a scoring signature and used 101-machine learning algorithms to establish and select the most effective prognostic model. The results showed that the signature constructed from these 28 genes has potential prognostic stratification value. Recent researches in investigating CAF-related signatures have highlighted their prognostic and therapeutic potential across cancer types. For instance, Zhang et al. previously developed a prognostic risk score model based on cancer-associated fibroblasts (CAFs) signatures in cervical cancer 41. Although our study shares methodological similarities with theirs study, we have employed machine learning algorithms to identify the model with optimal performance during the construction of the scoring system, as opposed to the conventional LASSO model. Similarly, analogous studies have been reported in ovarian cancer, further corroborating the applicability of this methodological framework 42. In contrast, our study has achieved a precise characterization of the microenvironment of eCAFs and SPP1+ macrophages in LUAD by integrating multi-omics data and combining the crosstalk between specific CAF subpopulations and SPP1+ macrophages. This advancement is crucial for understanding the local stromal-immune interaction.

To further illustrate the superiority of the model, the researchers selected several clinically recognized immune checkpoints, prognostic biomarkers as well as markers previously reported to be associated with LUAD prognosis to draw time-dependent ROC curves 43, 44. We discovered that this model has a more remarkable performance at 1-, 3-, and 5-year survival time points (Supplementary Figure 4).

In summary, this study identified eCAFs closely associated with poor prognosis in lung adenocarcinoma, characterized eCAFs' role in ECM remodeling via extracellular matrix proteins, and explored the interactions between eCAFs and SPP1+ macrophages, including potential ligand-receptor pairs which provided potential targets for targeting eCAFs therapy. Furthermore, we derived eCAFs-related signatures, offering novel tools and strategies for clinical prognostic stratification. However, our study has certain limitations. First, the samples are still insufficient and need to be expanded. Second, our study lacks in vivo and in vitro experimental validation, and we plan to supplement experiments in the future to make our conclusions more reliable. Third, the prognostic stratification model we built requires validation with more datasets and real clinical cohorts. Meanwhile, although the model demonstrates acceptable generalizability across independent LUAD datasets, its performance remains at a moderate level. Fourth, while the CIBERSORTx deconvolution algorithm may not be entirely precise, it remains the mainstream method for data processing. At the same time, as a highly interactive system, the tumor microenvironment involves intricate interactions among its components. While our study highlights eCAFs and SPP1+ macrophages as key drivers, their roles are likely intertwined with other TME constituents. This complexity is acknowledged as another limitation and we propose that our findings serve as a foundational framework for future research to dissect intercellular interactions in greater detail of the TME.

While our study establishes a robust association between eCAF-SPP1+ macrophages interactions and LUAD progression through multi-omics integration, we acknowledge that the correlative nature of bulk transcriptomic analyses inherently limits causal inference. Nonetheless, these limitations mentioned above do not undermine the overall reliability of our findings. Based on the findings of multi-omics studies, verifying the interactions between the matrix and the immune system through experimental systems will be the focus of subsequent research. This includes but is not limited to constructing orthotopic LUAD animal models, analyzing the microenvironment remodeling induced by treatment through longitudinal spatial transcriptomics, screening with high-throughput organoids to determine combined targeting strategies, and validating signaling pathways in vitro, among other experiments. We have preliminarily established the prognostic significance of the interaction between eCAFs and SPP1+ macrophages. We believe that our study can provide new insights for the further exploration of the crosstalk between fibroblasts and macrophages.

## Supplementary Material

Supplementary figures and tables.
