# Supplementary material for: Integration of Multi-Scale Profiling and Machine Learning Reveals the Prognostic Role of Extracellular Matrix-Related Cancer-Associated Fibroblasts in Lung Adenocarcinoma
Source: Int J Med Sci. 2025 Jun 12;22(12):2956–72. doi: 10.7150/ijms.113580 (PMC12243870; doi:10.7150/ijms.113580)
Supplement: Supplementary file 1 — Supplementary figures and tables. [file ijmsv22p2956s1.pdf]

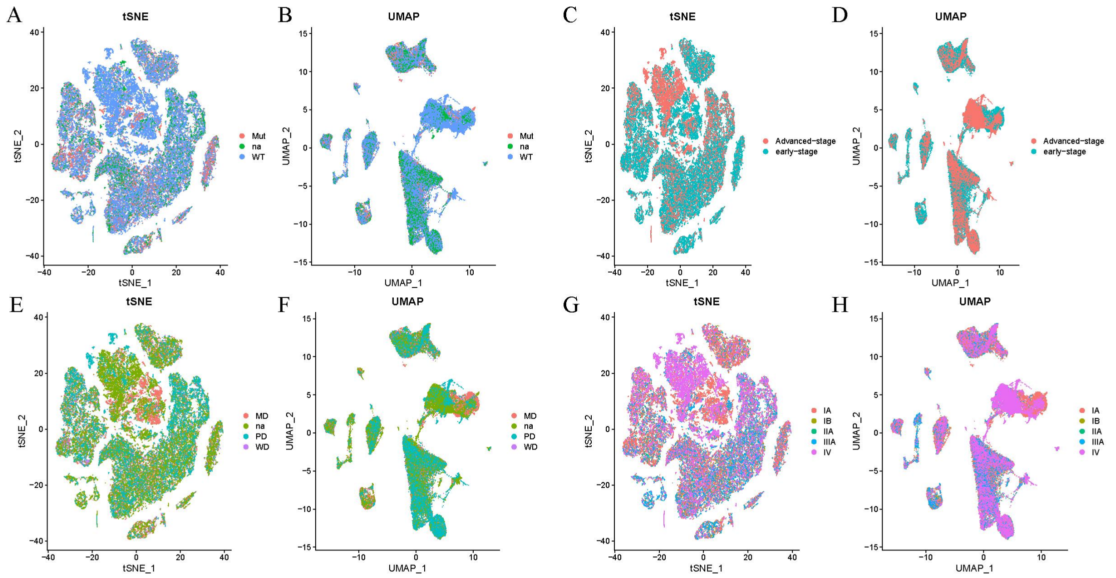

Supplementary Figure 1 Dimensionality reduction maps across different features. t-Distributed Stochastic Neighbor Embedding (t-SNE)-based dimensionality reduction map and Uniform Manifold Approximation and Projection (UMAP)-based dimensionality reduction map in EGFR-mutations (A-B), stages (C-D), pathological classifications (E-F) and TNM (G-H).

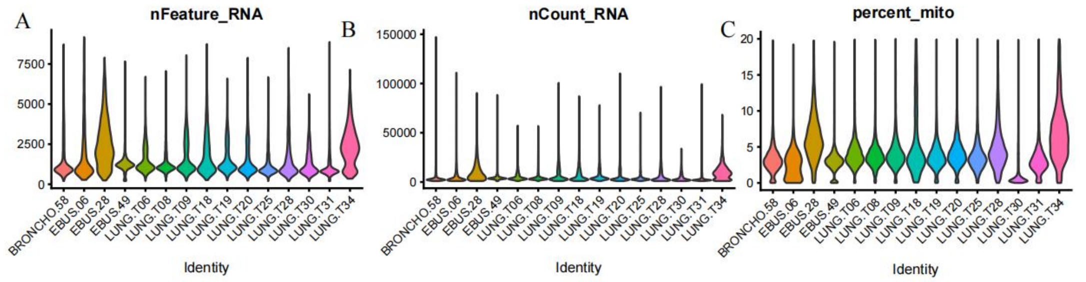

Supplementary Figure 2 The schematic of the quality control standards used for the single-cell data in the study. (A) Genes detected per cell were set to ≥ 200 and ≤ 10,000. (B) Total number of RNA reads detected per cell was set to ≥ 100 and ≤ 150,000. (C) The proportion of reads to mitochondrial genes was set to ≤ 20%.

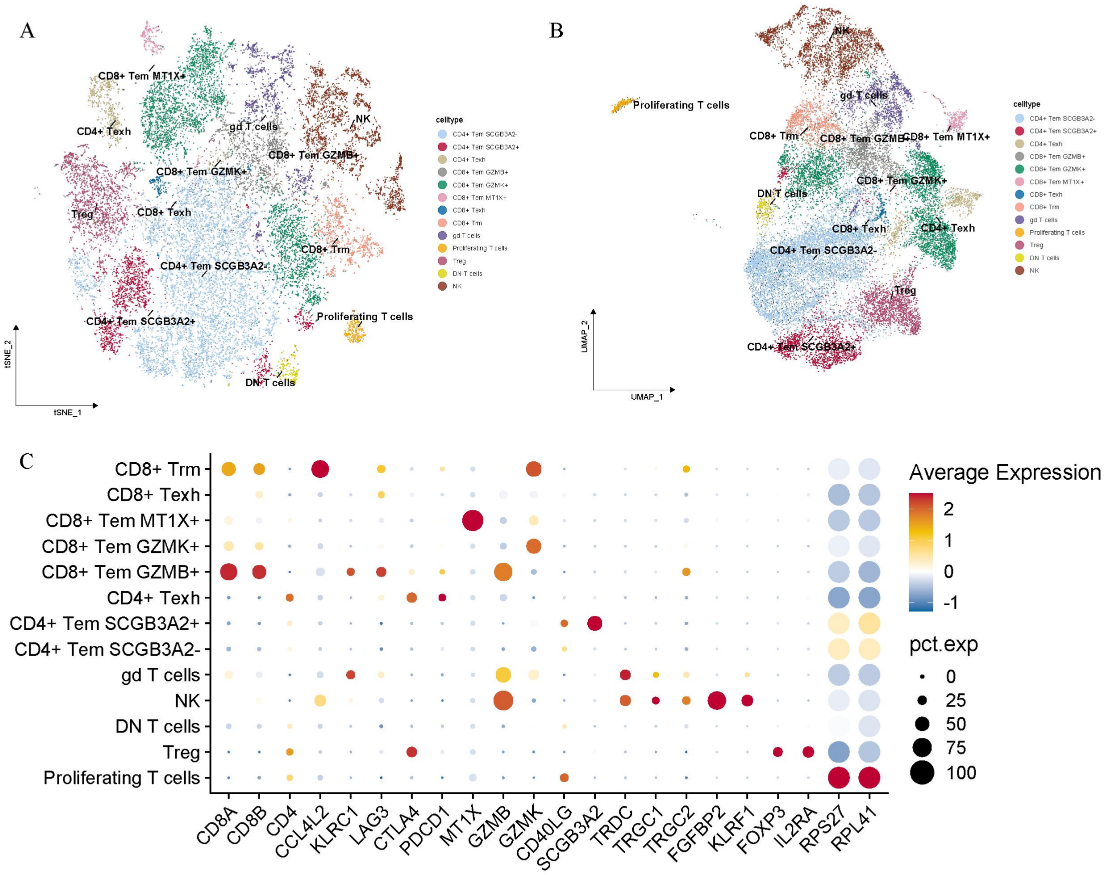

Supplementary Figure 3 Detailed annotation of T cells subpopulations. (A) tSNE-based dimensionality reduction map of T cells. Different cell clusters are represented by distinct colors. (B) UMAP-based dimensionality reduction map of T cells. (C) Dotplot showing the expression of representative marker genes. The color represents the average expression level, and the dot size indicates the percentage of cells expressing the gene.

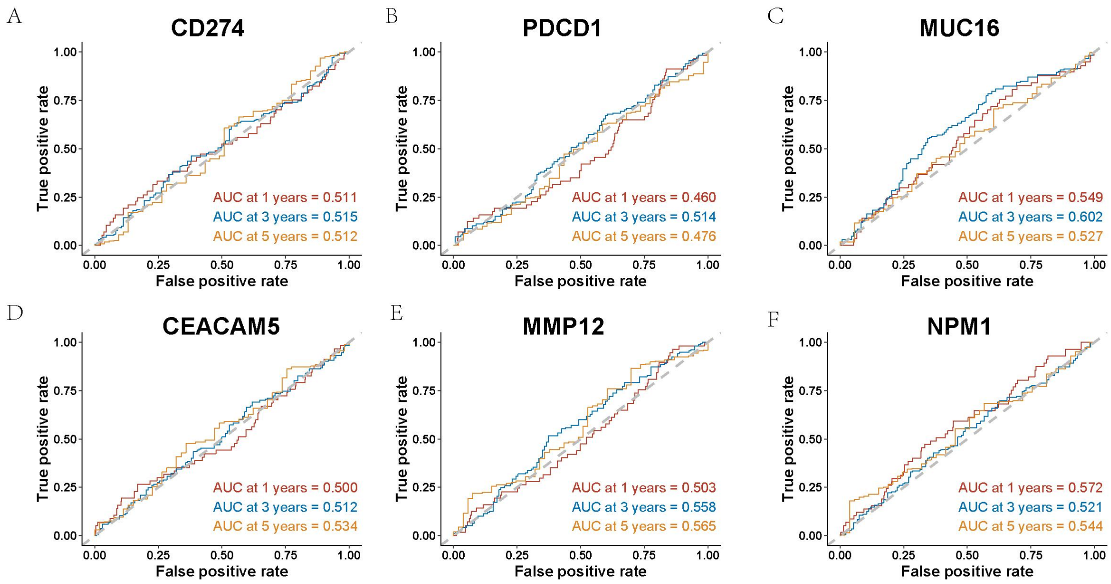

Supplementary Figure 4 The comparison of the model performance with existing

LUAD prognostic biomarkers.

Supplementary Table1. The association between the infiltration abundance of eCAFs and racial differences

Variables	Total (n = 503)	High (n = 251)	Low (n = 251)	p=0.751
race.demographic, n (%)				
american indian or alaska native	1 (0)	1 (0)	0 (0)	
asian	8 (2)	4 (2)	4 (2)	
black or african american	52 (10)	23 (9)	29 (12)	
not reported	54 (11)	25 (10)	29 (12)	
white	388 (77)	198 (79)	189 (75)	

Supplementary Table 2. The association between the infiltration abundance of SPP1+Macrophages and racial differences

Variables	Total (n = 503)	High (n = 251)	Low (n = 251)	p=0.898
race.demographic, n (%)				
american indian or alaska native	1 (0)	0 (0)	1 (0)	
asian	8 (2)	5 (2)	3 (1)	
black or african american	52 (10)	27 (11)	25 (10)	
not reported	54 (11)	26 (10)	28 (11)	
white	388 (77)	193 (77)	194 (77)	
